# JAK-STAT Signaling and Beyond in the Pathogenesis of Spondyloarthritis and Their Clinical Significance

**DOI:** 10.1007/s11926-024-01144-x

**Published:** 2024-03-16

**Authors:** Siba P. Raychaudhuri, Ruchi J. Shah, Sneha Banerjee, Smriti K. Raychaudhuri

**Affiliations:** 1 Department of Rheumatology, UC Davis Medical Center, Sacramento, CA USA; 2 VA Sacramento Medical Center, Department of Veterans Affairs, Northern California Health Care System, Mather, CA USA; 3 UC Davis School of Medicine, Davis, CA USA

**Keywords:** SpA, JAK-STAT, Ankylosing spondylitis, Signaling molecules

## Abstract

**Purpose of Review:**

Janus kinase–signal transducers and activators of transcription cell signaling proteins (JAK-STATs) play a key regulatory role in functioning of several inflammatory cytokines. JAK-STAT signaling proteins are the key regulators of the cytokine/cytokine receptor system involved in the pathogenesis of various autoimmune disease including spondyloarthritis (SpA). This article mainly highlights the JAK-STAT signaling system, its association with the relevant cytokine/cytokine-receptor system, and its regulatory role in pathogenesis of SpA. Also, we have briefly addressed the principle for the use JAKi in SpA and the current status of use of JAK inhibitors (JAKi) in SpA.

**Recent Findings:**

Recent developments with newer JAK molecules as well as other molecules beyond JAK inhibitors are now an exciting field for the development of novel therapies for autoimmune diseases and various malignant conditions. In this article, we have provided a special emphasis on how various cell signaling systems beyond JAK/STAT pathway are relevant to SpA and have provided a comprehensive review on this upcoming field in respect to the novel TYK2 inhibitors, RORγT inhibitors, mTOR inhibitors, NGF inhibitors, and various STAT kinase inhibitors.

**Summary:**

SpA are a group of autoimmune diseases with multifactorial etiologies. SpA is linked with genetic predisposition, environmental risk factors, and the immune system-mediated systemic inflammation. Here, we have provided the regulatory role of JAK/STAT pathway and other intracellular signaling system in the pathogenesis of SpA and its therapeutic relevance.

## Introduction

Janus kinase–signal transducers and activators of transcription cell signaling proteins (JAK-STATs) play a crucial role in pathogenesis of several autoimmune conditions. The Janus kinases, also known as JAKs, are a group of intracellular molecules involved in functioning of several cytokine molecules. These molecules have a prominent role in adaptive and innate immunity as well as hematopoiesis rendering them as targets for therapeutic medicines in inflammatory and myeloproliferative diseases. The discovery of JAK2 mutations first indicated that abnormal JAK-STAT signaling kinase system could be the pivotal feature in the disease process of Philadelphia-negative myeloproliferative neoplasms. This idea led to the development of JAK inhibitors, and currently, there is a rapid surge in clinical development of several inhibitors targeting the JAK-STAT pathway. Among all the JAK inhibitors (JAKi), even though the goal is to target the adenosine triphosphate binding site within the kinase domain, different JAKis differ in their specificity of targeting specific JAKs. For example, upadacitinib mainly targets JAK1 while ruxolitinib targets JAK1 and JAK2. Thus, the newer generation JAKis are more specific and are expected to be associated with fewer side effects. This article mainly highlights the role of JAK-STAT signaling pathway in pathogenesis of spondyloarthritis (SpA) and the role of JAKi in treatment of SpA. We will also briefly discuss the newer drugs in pipeline, beyond JAKis which provide a whole new spectrum of molecules for treatment of several autoimmune diseases with fewer side effects.

## Understanding the Basics of JAK-STAT Pathway

**Fig. 1:**
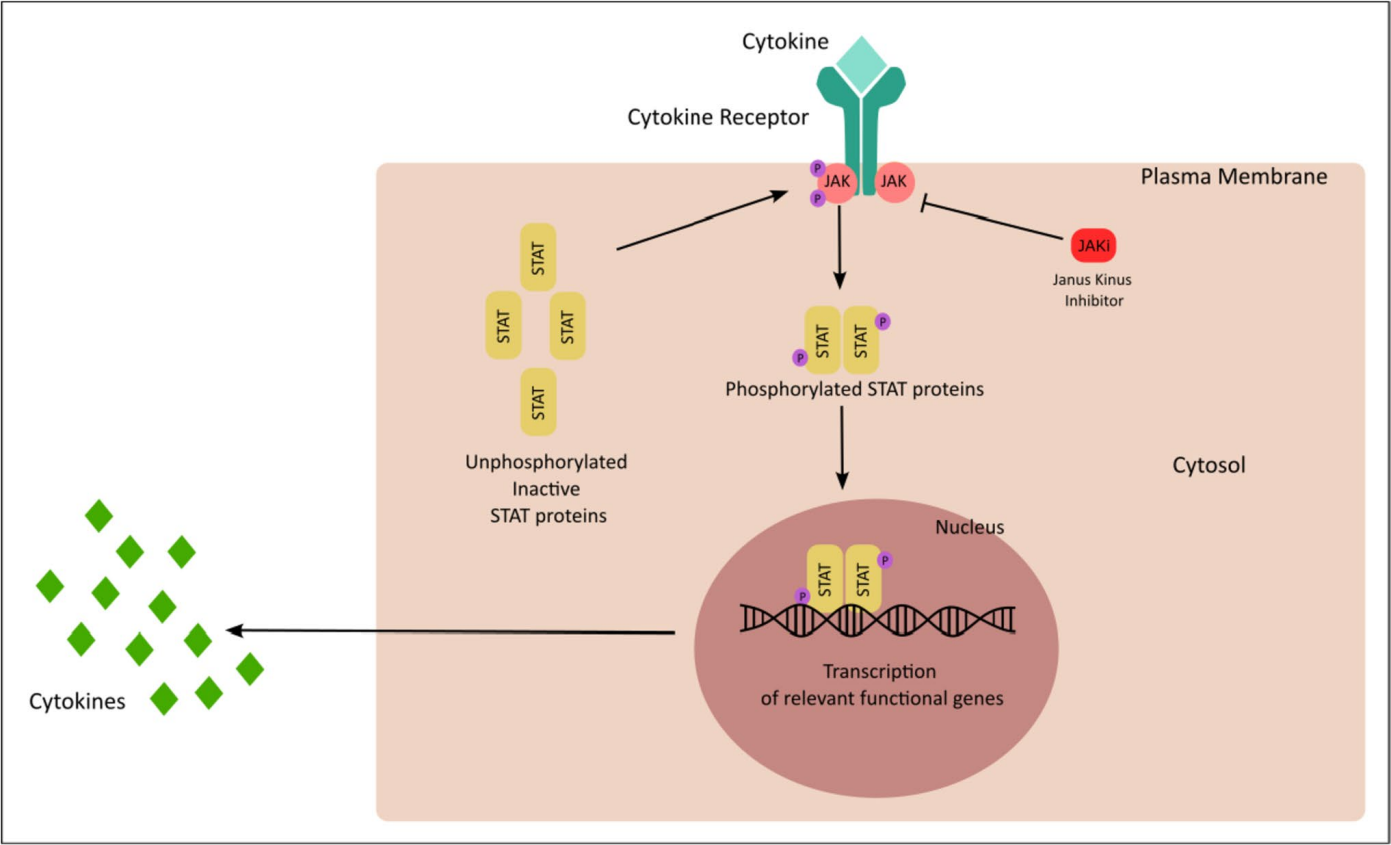
JAK/STAT signaling sequences for immune response and inflammation. The JAK/STAT signaling pathway, activated by the cytokine/cytokine receptor systems and growth factors, is an established processes for immune response, T cell proliferation, and T cell apoptosis. Interaction of the cytokine/cytokine receptor system induces to conformational changes in its intracellular domain, which leads to phosphorylation of intracellular JAK proteins. Phosphorylated JAKs lead to activation/phosphorylation and dimerization of STATs which then move as homo/hetero dimers into the nucleus and bind to specific DNA binding sites. Thus, JAK/STAT signaling proteins induce gene transcription and cytokine production and play a critical role in the inflammatory/proliferative cascades of various inflammatory/autoimmune diseases

**Fig. 2:**
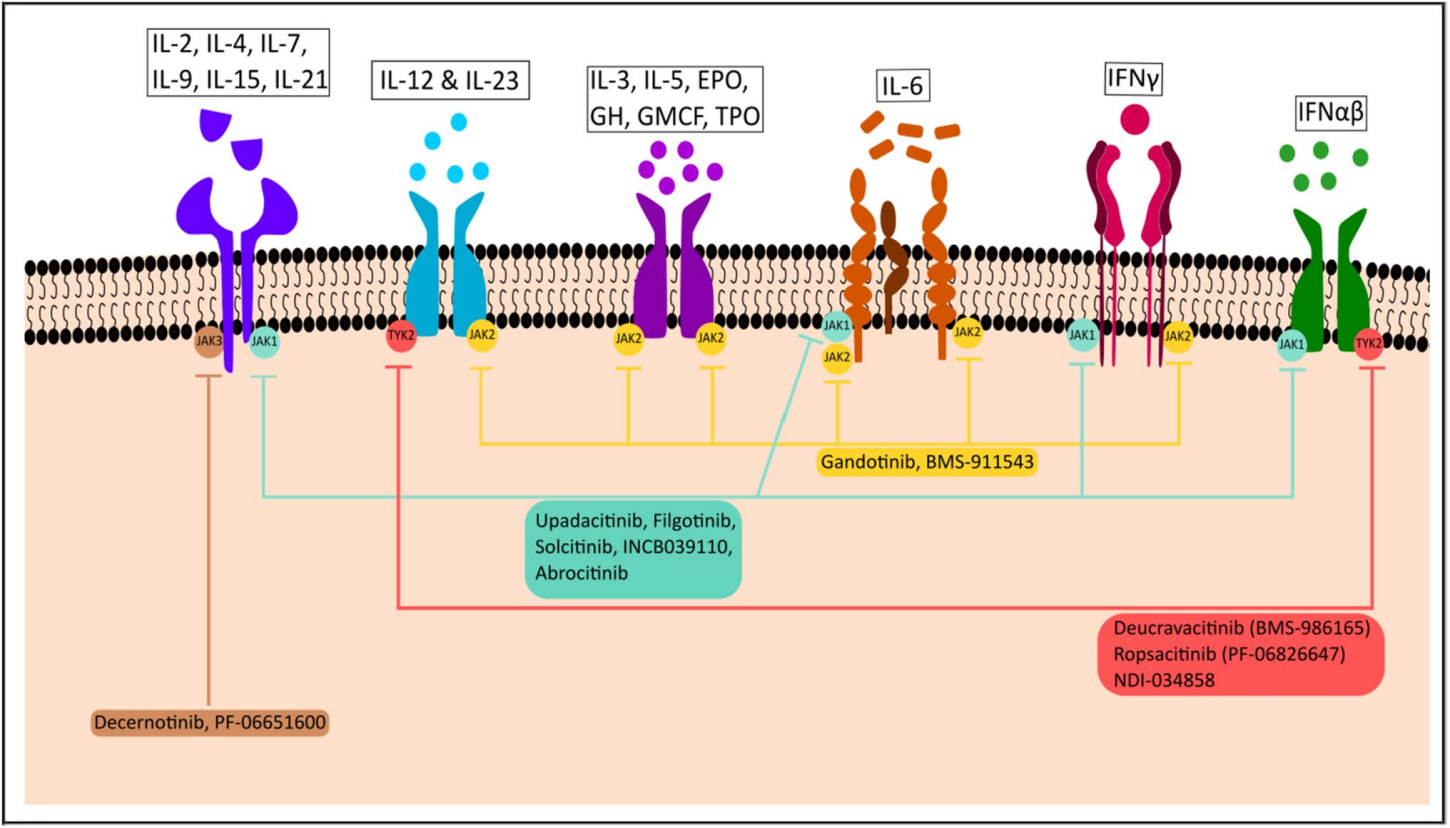
JAK STAT signaling proteins in spondyloarthropathy and possible mechanisms of action of the JAK inhibitors. As illustrated in this Fig. 2 and the Table 1, various inflammatory cytokines including interferons, interleukins, interferon-like cytokines, growth factors, and colony-stimulating factors; bind to their specific receptors resulting in activation of specific JAK-STAT pathways. Specifically, IL-2, IL-9, IL-12, IL-21, and IL-23 are well known to have contributing roles in the pathogenesis of spondyloarthritis (SpA). It also demonstrates how the new generation of JAK inhibitors can block a specific JAK kinase and thus can be used for treatment of SpA

The JAK-STAT pathway is a central axis involved in the inflammatory response and carcinogenesis. The pathway includes several cytokines, transmembrane receptors, JAK proteins (JAK1, JAK2, JAK3, and TYK2), and STAT proteins (STAT1,2,3,4,5,5a, and 6) [1, 2•, 3, 4•, 5]. As illustrated in Figs. 1 and 2, the first step in the functioning of JAK STAT pathway involves the binding of several cytokines to a transmembrane receptor. The pathway is triggered by binding of several cytokines like interferons, growth factors, hormones, and interferon-like cytokine to their respective cell surface receptors. This in turn activates the JAK molecules associated with the intracellular component of these receptors followed by subsequent dimerization phosphorylation of their tyrosine residues on the catalytic domain of the receptors. Next, the SH2 domain of the STAT protein docks on the phosphorylated tyrosine residues leading to phosphorylation of STAT proteins. Lastly, the STAT dimers translocate to the nucleus to regulate gene transcription by associating with DNA-binding sites. In this way, the JAK-STAT proteins potentiate the action of several inflammatory cytokines involved in the pathogenesis of several autoimmune conditions including ankylosing spondylitis, psoriasis, rheumatoid arthritis, and inflammatory bowel disease [6].

## Role of JAK-STAT Signaling System in Spondyloarthritis

The JAK/STAT signaling pathway, activated by the cytokine/cytokine receptor systems and growth factors, is an established processes for immune response, T cell proliferation, and T cell apoptosis. These in turn provides the regulatory role of the cytokine/cytokine receptor system in the pathogenesis of multiple autoimmune conditions [1, 2•, 3, 4•, 7–10] (Figs. 1 and 2). JAK STAT signaling system regulated key functions of certain cytokines associated in the pathogenesis of SpA are mentioned below:

- IL-2: promotes proliferation and differentiation of effector and memory cells and also promotes regulatory T cell development
- IL-9: important for survival and activation of T cells
- IL-12: induces Th1 cell differentiation
- IL-22: stimulates epithelial cell proliferation and production of other inflammatory cytokines and chemokines
- IL-23: induces Th17 cell differentiation and expansion
- Interferon gamma: involved in functioning of Th1 cells

Because of the signaling cross-talks between the JAK-STAT pathway and specific cytokines mentioned above, described in Fig. 2, it is expected that the JAK-STAT signaling kinase proteins are of major importance in the pathogenesis of SpA. Polymorphisms of JAK-STAT kinases may be a plausible mode of mechanism in the etiology of SpA diseases. JAK2 polymorphisms have been reported to be associated with AS in a study conducted in Chinese Han population; here, a possible relationship of JAK2 and STAT3 polymorphisms was investigated in patients with AS [11]. In this study, genotype markers of JAK2 or STAT3 did not contribute to the susceptibility of AS; however, the study did find an association of a haplotype in JAK2 locus with AS. We have reported similar supportive evidence that interleukin 23 (rIL-23) induces phosphorylation of JAK2 and STAT3 in activated CD3^+^ T cells in PBMC of psoriatic arthritis (PsA) patients. Moreover, we noticed that tofacitinib significantly inhibited phosphorylation of JAK2 and STAT3. Also, tofacitinib did inhibit IL-23-induced proliferation of the IL-17^+^ TEM cells. These novel findings provide new insights for the pathogenesis SpA that generation of the pathologic IL-17^+^ TEM cells and their proliferation are regulated by the JAK-STAT signaling proteins [12••].

Several animal studies have been conducted to study the role of JAK-STAT signaling pathway in the pathogenesis of autoimmune disease, including SpA. As we know, SpA is an autoimmune disease affecting skin, enthesis, and peripheral and axial joints. It includes ankylosing spondylitis, psoriatic arthritis, reactive arthritis, enteropathic arthritis, and undifferentiated SpA. In one of the studies, a statistically significant reduction (*p* < 0.05) were noted in the disease activity scores for histological and clinical inflammation in SKG mice after treatment with JAKi [13]. Gracey et al. demonstrated that the progression of SpA in a mouse model can be inhibited by a potent and specific TYK2 inhibitor—NDI031407A. It was further noted that selective TYK2 inhibition impacts the IL-23 pathway which is essential in the pathogenesis of SpA. Model NDI 0310407 was noted to halt joint space narrowing, bone marrow edema, and enthesitis-related synovitis on MRI in IL-23 mini circle model [14]. Another experimental study demonstrated the effect of oral JAKi lavage in suppressing inflammation and inhibiting the periosteal bone formation in study animals. Further studies have demonstrated that a few signal nucleotide polymorphisms (SNPs) as well as silencing RNA suppress TYK2 molecules and thus inhibiting the function of several inflammatory cytokines and thus providing a promising target for treatment of several immune-mediated disease including SpA. Former studies have demonstrated that loss of function of TKY2 SNP was associated with less severe disease manifestations of SpA mainly noted as lower rates of spinal fusion in these patients [14].

With respect to the immunomodulatory role of the abovementioned IL-2, IL-9, IL-12, IL-22, and IL-23 cytokines in the pathogenesis of SpA and its association with the JAK-STAT signaling systems as mentioned above, it has been substantiated by several studies in animal models and in human [10, 11, 12••, 14]. Among these cytokines, IL-2 is a well-known growth factor for T cells and has a broad regulatory role in array of T cell mediated diseases including SpA [3–9]. IL-2-induced T cell growth is mediated through pan JAK activation (JAK1, JAK2, and JAK3) [3–9] that provides explanation for efficacy of tofacitinib (pan jak inhibitor), upadacitinib (JAK1 inhibitor), and filgotinib (JAK1 inhibitor) in PsA and AS (Table 3). IL-9 is a growth factor for T cells and both IL-9 and lL-22 regulate inflammatory cascades of SpA [5, 10] and both IL-9 and IL-22 activates JAK1 so specific JAK1 inhibitors like upadacitinib and filgotinib (Table 3) counters JAK1 activation in AS and PsA. IL-23 regulates Th17 cells and promotes secretion of IL-17 and IL-22 through activation of JAK2 and TYK2 [11, 12••] 1) which are important for the disease process of psoriasis and PsA so tofacitinib (pan JAKi, which includes JAK2 inhibition) and deucravacitinib (TYK 2 inhibitor) are likely countering IL-23 activation by inhibiting JAK2 and TYK2, respectively, and effective in psoriasis and PsA (Table 3 and 1). It is worth mentioning that anti-IL23 mab is effective for psoriasis and PsA and does not work for AS. It is likely the two drugs tofacitinib and upadacitinib approved for AS are working by inhibiting the CD3 T cells, Th9 cells, and IL-22.

**Table 1:** First-generation and newer generations of JAK-STAT inhibitors and their clinical uses

	JAK inhibitors	Target ​	Indications	Current approval status
First Generation​ JAKi	Baricitinib​	JAK1/JAK2​	Rheumatoid arthritis, alopecia areata, COVID-19	FDA approved
	Ruxolitinib​	JAK1/JAK2​	Atopic dermatitis and vitiligo (topical), polycythemia vera, myelofibrosis, graft versus host disease	FDA approved
	Tofacitinib​	JAK1, JAK2, JAK3​	Rheumatoid arthritis, psoriatic arthritis, ankylosing spondylitis, ulcerative colitis, COVID-19	FDA approved
Newer generation JAKi	Upadacitinib	JAK1 > JAK2, JAK3​	Rheumatoid arthritis, psoriatic arthritis, ankylosing spondylitis, nr-axSpA Atopic dermatitis Ulcerative colitis Crohn disease	FDA approved
	Abrocitinib	JAK1	Moderate to severe atopic dermatitis	FDA approved
	Pacritinib	JAK2 > JAK3, TYK2	High-risk myelofibrosis	FDA approved
	Deucravacitinib (BMS-986165)	TYK2	Moderate to severe chronic plaque psoriasis	FDA approved
	Fedratinib	JAK2	Myelofibrosis	FDA approved
	Filgotinib GLPG0634​	JAK1 > JAK2​	Rheumatoid arthritis (approved by EMA)	EMA approved
	Solcitinib GSK2586184​	JAK1​	Moderate to severe plaques psoriasis, moderate to severe ulcerative colitis	Currently under trial
	Decernotinib (VX-509)	JAK3	Rheumatoid arthritis	Currently under trial
	Itacitinib (INCB039110​)	JAK1 > JAK2​	Lymphoma	Recently completed Phase III trial
	Ochromycinone (STA-21)​	STAT3​	Topical drug in psoriasis	Completed Phase II trial
	PF-06700841​	TYK2/JAK1​	Moderate to severe plaque psoriasis	Completed Phase IIa trial
	PF-06651600​	JAK3​	Alopecia areata	Completed Phase III trial
	NDI-034858-TYK2	TYK2	Psoriasis (moderate/severe) and psoriatic arthritis	Completed Phase IIb trial

**Table 2:** Clinical indications of JAK inhibitors and TYK2 inhibitor in psoriatic arthritis

JAK inhibitors: current status in psoriatic arthritis	JAK isoforms inhibited	Dose for PsA	Approval status	ACR20 response in PsA (references)
Tofacitinib	JAK3 > JAK1,JAK2 > TYK2	5 mg twice daily 11 mg daily (extended release tablets)	Approved by FDA (2017)	~ 60% at wk 52 (46)
Upadacitinib	JAK1	15 mg once daily	Approved by FDA (2021)	~ 70% at wk 12 (47)
Deucravacitinib	TYK2	6 mg once daily	Trials (Phase IIb) for PsA are in progress Approved by FDA for psoriasis (2022)	~ 50% at wk 16 (48)
Filgotinib	JAK1	200 mg once daily	Not yet approved by FDA for PsA; still in trial	~ 80% at wk 16 (49)

## Introduction to JAK STAT Inhibitors

JAK inhibitors are novel small molecules which are termed as targeted synthetic disease modifying anti-rheumatic drugs (ts-DMARDs). First-generation JAKi are non-selective and inhibit multiple JAK isoforms, whereas, the newer generation JAKi selectively inhibit certain specific JAK isoforms, such as JAK-1, JAK-2, JAK-3, and TYK2 (Table 3 and Fig. 2).

### Classification of JAK Inhibitors

First-generation JAKi includes ruxolitinib, tofactinib, and baricitinib.

While ruxolitinib was the first JAK inhibitor to be approved by FDA, it is only approved for polycythemia Vera and does not affect in SpA. Among the first-generation JAKi, tofacitinib, non-selective JAKi (JAK1, 3 > JAK2, and TYK2), has been studied and noted to be effective in SpA.

Newer generation JAKi include the more selective JAKi inhibiting specific isoforms of JAK1, JAK2, JAK3, and TYK2 (Table 3). Given more directed and specific inhibition of JAK isoforms, these JAKi are expected to be associated with fewer side effects. Upadacitinib (selective JAK1 inhibitor) and filgotinib (JAK1 inhibitor) have been studied for SpA treatment. Filgotinib is a highly selective JAK1 inhibitor approved for use in Europe and Japan for Rheumatoid Arthritis. It has not been yet approved by FDA. Various other investigational molecules have been studied for other immune-mediated diseases [5].

### Clinical Efficacy of JAK Inhibitors in Spondyloarthritis

The purpose of this article is to provide an overall view of the regulatory role JAK/STAT signaling system in the disease processes of SpA and its clinical impact. So here, we will also provide a brief review on efficacy and safety of JAKi in SpA.

Clinical trials have been carried out to determine the efficacy of tofacitinib, upadacitinib, and filgotinib in SpA—mainly ankylosing spondylitis and psoriatic arthritis. For ankylosing spondylitis, all the study patients were noted to have inadequate response to NSAIDs and a comparison was made among patients receiving JAKi versus placebo by measuring clinical outcomes in the form of disease activity as ASAS20, ASAS40, and BASDAI50 as well as improvement in quality of life and resolution of inflammation on imaging. SELECT-AXIS1 trial studied the efficacy and safety of upadacitinib in patients with ankylosing spondylitis who are naïve to biologic DMARDs [15]. It was a multicenter, randomized, double-blind placebo-controlled trial which was done over two phases. First phase spanned over 14-week period and randomized the participants 1:1 to upadacitinb and placebo. At the end of week 14, patients receiving upadacitinb showed definite improvement in disease activity measured as ASAS40 and ASDAS scoring as well as MRI scoring for disease activity. Interim follow-up of this trial demonstrated safety of the molecule over a course of one year. SELECT-AXIS 2 trial evaluated the efficacy of Upadacitinib in non-radiographic SpA. It demonstrated 22% improvement in ASAS40 response at week 14 while comparing participants receiving upadacitinib versus placebo [16]. Upadacitinib demonstrated efficacy of JAKi in treatment of radiographic as well as non-radiographic axial-SpA. In a latter report, Baraliakos et al. have reported the 52 weeks efficacy/safety in AS patients who had inadequate responses to biologic disease-modifying antirheumatic drugs (bDMARD-IR) from the SELECT-AXIS 2 study. In this study, AS patients demonstrated sustained improvement with upadacitinib 15 mg/daily up to 52 weeks in the bDMARD-IR patients. Efficacy was overall similar in patients who had lack of efficacy or intolerance to bDMARDs and prior use of TNFi versus IL-17i exposure [17].

Being a first-generation JAK inhibitor, tofacitinib inhibits multiple JAK isoforms as discussed above and have been noted to be effective in treating SpA. A phase III clinical trial has demonstrated significant efficacy of tofacitinb in radiographic SpA. In this study, the ASAS20 response rate was significantly (*p* < 0.0001) greater with tofacitinib (56.4%; 75 of 133) versus placebo (29.4%; 40 of 136), and the ASAS40 response rate was also significantly (*p* < 0.0001) greater with tofacitinib (40.6%; 54 of 133) versus placebo (12.5%; 17 of 136) [18].

Lastly, a double blind, placebo-controlled phase two study (TORTUGA) compared the disease activity in patients with radiographic SpA with inadequate response to NSAIDs receiving filgotinib versus placebo at week 12. It demonstrated greater improvement in ASDAS score as well as ASAS 20 and ASAS40 in filgotinib arm as compared to placebo [19].

Because of space limitation, we have provided the current status of clinical use of JAKi and TYK2 inhibitor in psoriatic arthritis in the Table 1.

### Safety Profile of JAK Inhibitors

These small molecules are noted to be relatively safe and have similar safety profile as compared to conventional DMARDs.

Given its impact on mounting immune response through production of cytokines, similar to cDMARDS, JAKi have been noted to be associated with increased risk of infections—most notably upper respiratory tract infections as well as urinary tract infections but more importantly even some serious infections like tuberculosis and herpes zoster infections [ 20••, 21–28]. Hence, patients are screened for chronic tuberculosis infection prior to initiation of JAKi. It is also recommended to vaccinate these patients against herpes zoster prior to initiation of therapy to prevent serious infection if exposed.

Esophageal candidiasis is another severe infection associated with JAKi leading to significant discomfort and morbidity in these patients.

Given JAK inhibitors can cause leucopenia and neutropenia, it is recommended to monitor complete blood count periodically while on JAKi as discussed above.

In terms of gastrointestinal side effects, JAKi can causes elevation in liver enzymes, nausea, and vomiting but most important complication is gastrointestinal perforation which is mainly seen in patients on concomitant NSAIDs or steroid therapy.

While there are no absolute contraindications for JAK inhibitor, cautious use is recommended in the setting of an active infection, absolute neutropenia < 1000/mm^3^ or absolute lymphopenia < 500/mm^3^. Cautious use is recommended in patients with severe renal and hepatic impairment. It is to be avoided in the patients with history of prior hypersensitivity reaction. Limited data is available regarding the safety of JAKi in pregnant and breast-feeding patients. Given prior studies of increased risk for thromboembolic events as well as major cardiovascular events (MACE) in patients with prior history of a cardiac event, JAKi use is not preferred [29]. The published results of the post-marketing ORAL Surveillance study (ORALSURV) which compared the JAKi tofacitinib with anti-TNF therapy in patients with rheumatoid arthritis (> 50 yrs age) who had cardiovascular risk factors has reported more frequent occurrence of cardiovascular and cancer adverse events with tofacitinib than with TNFi. These observations have led to changes in the recommendations for the use of JAK inhibitors. Subsequent to this study, the FDA extrapolated the ORALSURV data beyond tofacitinib to include baricitinib and upadacitinib. The FDA has recommended to use tofacitinib, baricitinib, and upadacitinib in patients who have had an inadequate response to TNF inhibitors or could not tolerate anti-TNF agents. Also, it has been recommended that the risks/benefits for patients with a history of smoking, and with risk factors for cardiovascular disease and malignancy should be considered prior to initiating/continuing tofacitinib, upadacitinib, or upadacitinib [30] (Table 2).

## Beyond JAK Inhibitor: Targeting the Cell Signal Molecules Beyond JAK Inhibitors for Treatment of SpA and Other Diseases

The success of JAK inhibitors in the treatment of SpA has opened a new avenue to target other critical cell signaling proteins for treatment of array of autoimmune disease. Here, we will briefly address this exciting prospective field of clinical immunology.

### Tyrosine Kinase 2 (TYK2) Inhibitors

**Fig. 3:**
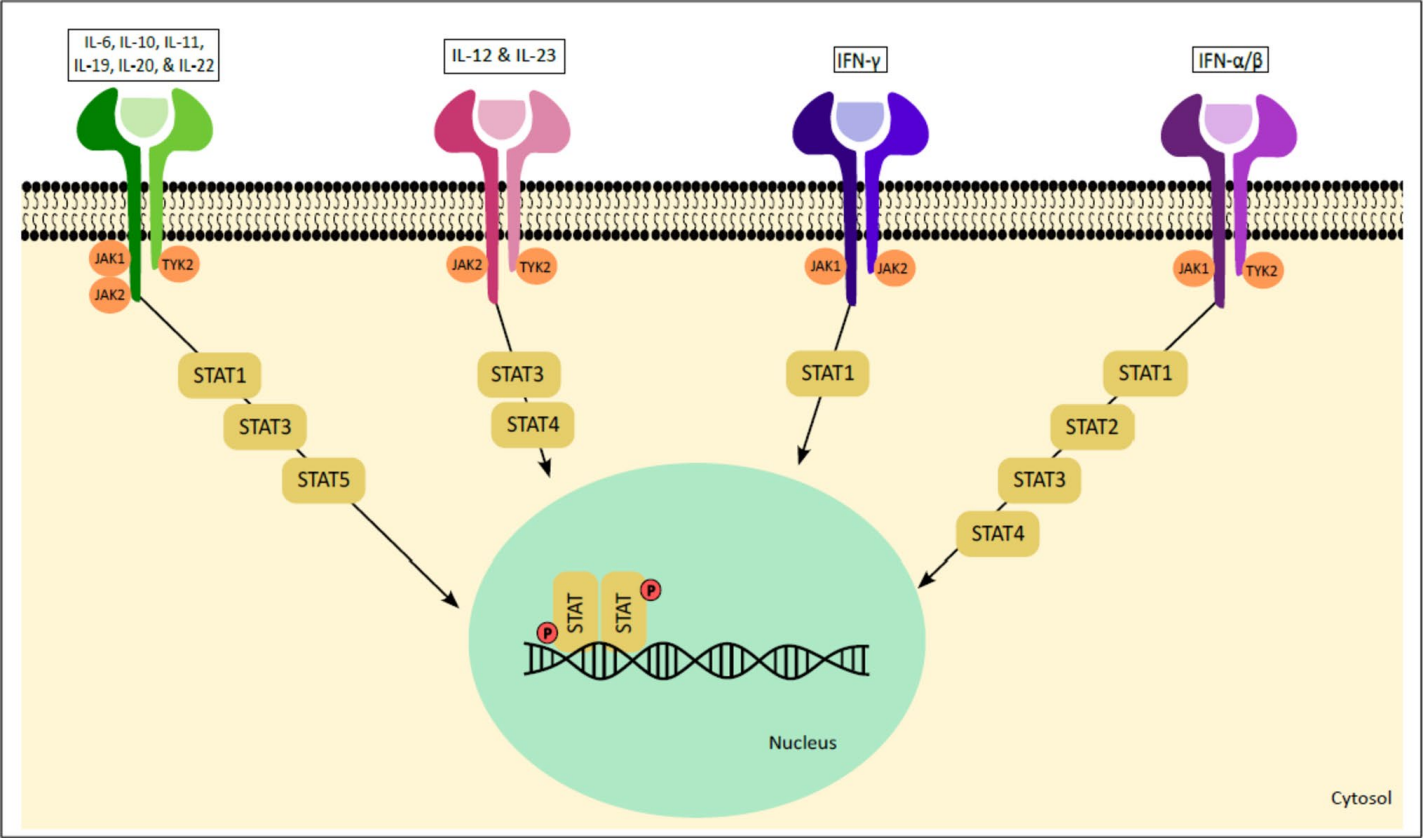
Tyk2 Signaling and its pathologic significance. Tyk2 mediates signaling of multiple cytokines as mentioned in this figure. Among these, IL-12, IL-22, and IL-23 are well known for their contributions in the disease process of spondyloarthritis (SpA). Seminal studies uncovering the basic science of TYK2 have provided sound foundations for targeting TYK2 in SpA and other related inflammatory diseases [31, 32••]

**TYK2**, an integral non-receptor tyrosine-protein kinase belonging to the JAK-STAT receptor family, orchestrates critical signaling pathways by engaging with ligands such as IL-6, IL-10, IL-12, IL-23, and type 1 IFNs, culminating in receptor dimerization and consequent activation of TYK2 as well as other members of the JAK family. Thus, TYK2 plays a multifaceted role, encompassing innate immune cell maturation, differentiation processes, and the modulation of immune responses relevant to inflammatory and autoimmune disorders (Fig. 3).

Seminal studies uncovering the basic science of TYK2 have provided sound foundations for targeting it in SpA and related inflammatory diseases [14, 31, 32••, 14]. Thus, TYK2 inhibitors may well be an excellent therapeutic option in the near future for SpA. So far, deucravacitinib is the only FDA-approved TYK2 inhibitor for treatment of psoriasis. Deucravacitinib is an allosteric inhibitor that binds to the pseudokinase JH2 (regulatory) domain of TYK2; this unique mechanism determines greater selectivity and a reduced risk of adverse events. Deucravacitinib became the first TYK2 inhibitor approved for the treatment of moderate-to-severe psoriasis [33].

Deucravacitinib is currently being evaluated for a number of SpA-associated diseases in phase 2 and 3 trials for psoriatic arthritis (NCT03881059), moderate to severe ulcerative colitis (NCT03934216), and Crohn’s disease (NCT04877990). A bright future can be expected for TYK2 inhibitors, with newer drugs and more indications.

### TrkA (Tropomyosin Receptor Kinase A) Inhibitor

Nerve growth factor (NGF), known for its crucial role in the development and survival of neurons, has recently emerged as a multifaceted agent with neuroprotective and anti-inflammatory roles. By inducing dimerization and autophosphorylation of the high-affinity receptor TrkA (tropomyosin receptor kinase A), NGF orchestrates a signaling cascade involving C-y1, Shc, FRS2, and PI3K. In a human, a novel function of NGF has been observed that along with TrkA induces proliferation and activation of T cells. NGF and NGF-R as a novel target have already been substantiated for psoriasis and PsA [34].This novel target is currently in development for a new class of drug for treatment of pain and immune dysregulation of psoriatic disease and other rheumatologic autoimmune diseases [34–36].

### mTOR Inhibitors

Mammalian target of rapamycin, or mTOR, is a kinase that takes part in the PI3-K/Akt/mTORC1 signaling cascade thought to regulate epidermal homeostasis, synovial cell proliferation, and T cell activation in psoriasis and PsA [37]. These cellular and molecular mechanisms along with series of observations of efficacy of rapamycin in psoriasis [38] and a recent report suggesting that in the HLA-B27 transgenic rat model of SpA, rapamycin inhibits arthritis and spondylitis support efforts to evaluate the efficacy of targeting the mTOR pathway in SpA patients [39].

### RORγt (Retinoic Acid Receptor-Related Orphan Receptor-γ) Inhibitors

In vivo and in vitro studies have demonstrated that RORγt inhibitors are helpful for the reduction of both skin and joint inflammation in suitable models of autoimmune skin and joint disease [40, 41]. Since RORγt is the transcription factor for Th17 cell differentiation, targeting RORγt is a very promising strategy and several small molecules targeting this have been prepared to treat Th17-mediated diseases such as psoriasis and PsA [40–42]. It is expected that RORγt may be a potential therapeutic agent for SpA and its related clinical conditions including psoriasis Table 3.

**Table 3:** Safety monitoring while on JAK inhibitors

Baseline investigations prior to initiation of JAK inhibitor	• Complete blood count with differential • Liver function test, kidney function test • Lipid panel • Rule out chronic infections including TB (QuantiFERON gold test), hepatitis B and C panel • Pregnancy test in women of reproductive age group
Lab monitoring while on JAK inhibitor therapy	Monitor the following labs 4–8 weeks after initiation of JAKi and every 3 months thereafter: • Complete blood count with differential to monitor for cytopenia • Liver function test Lipid panel to be checked 4–8 weeks after initiation of JAKi to monitor for hyperlipidemia

### STAT3 and STAT4 inhibitors

Studies using animal models have demonstrated that mice with CD4 + T cell-specific deletion of STAT3 exhibit defective TH17 differentiation and impaired development of experimental autoimmune encephalomyelitis (EAE) [43]. STAT4—in a collagen-induced arthritis (CIA) animal model, WT mice were more susceptible to CIA than were the STAT4-deficient mice. In a systemic scleroderma (SSC) animal model, the STAT4-deficient mice had a significant reduction in collagen accumulation and alpha-smooth muscle actin-positive myofibroblasts number [44]. Furthermore, in non-obese diabetic mice, STAT4 inhibition impedes the development of type 1 diabetes [45]. Therefore, STAT3/STAT4 are promising therapeutic target to treat human autoimmune diseases including SpA.

## Conclusion

Role of JAK inhibitors in the treatment of several autoimmune diseases including SpA is now well established. Both tofacitinib and updacitinib is now FDA approved for the treatment of psoriatic arthritis and ankylosing spondylitis; and in addition, upadacitinib is approved for non-radiographic axial spondyloarthritis** (**nr-axSpA). Ongoing research in the flied have led to the discovery of several selective and non-selective JAK inhibitors. With several ongoing trials, future beholds several molecules beyond JAK inhibitors, safety and efficacy of which seem to be promising but yet to be established. We have also addressed how various cell signaling systems beyond JAK/STAT pathway are relevant to SpA and have provided a brief review on this upcoming field.
